# cAMP-Dependent Synaptic Plasticity at the Hippocampal Mossy Fiber Terminal

**DOI:** 10.3389/fnsyn.2022.861215

**Published:** 2022-04-04

**Authors:** Meishar Shahoha, Ronni Cohen, Yoav Ben-Simon, Uri Ashery

**Affiliations:** ^1^Faculty of Life Sciences, School of Neurobiology, Biochemistry and Biophysics, Tel Aviv University, Tel Aviv, Israel; ^2^Sagol School of Neuroscience, Tel Aviv University, Tel Aviv, Israel; ^3^Department of Neurophysiology, Vienna Medical University, Vienna, Austria

**Keywords:** cAMP, PKA, synaptic plasticity, mossy fiber synapse, LTP, forskolin-induced potentiation

## Abstract

Cyclic adenosine monophosphate (cAMP) is a crucial second messenger involved in both pre- and postsynaptic plasticity in many neuronal types across species. In the hippocampal mossy fiber (MF) synapse, cAMP mediates presynaptic long-term potentiation and depression. The main cAMP-dependent signaling pathway linked to MF synaptic plasticity acts via the activation of the protein kinase A (PKA) molecular cascade. Accordingly, various downstream putative synaptic PKA target proteins have been linked to cAMP-dependent MF synaptic plasticity, such as synapsin, rabphilin, synaptotagmin-12, RIM1a, tomosyn, and P/Q-type calcium channels. Regulating the expression of some of these proteins alters synaptic release probability and calcium channel clustering, resulting in short- and long-term changes to synaptic efficacy. However, despite decades of research, the exact molecular mechanisms by which cAMP and PKA exert their influences in MF terminals remain largely unknown. Here, we review current knowledge of different cAMP catalysts and potential downstream PKA-dependent molecular cascades, in addition to non-canonical cAMP-dependent but PKA-independent cascades, which might serve as alternative, compensatory or competing pathways to the canonical PKA cascade. Since several other central synapses share a similar form of presynaptic plasticity with the MF, a better description of the molecular mechanisms governing MF plasticity could be key to understanding the relationship between the transcriptional and computational levels across brain regions.

## Cyclic Adenosine Mono-Phosphate-And Protein Kinase A-Dependent Mechanisms of Synaptic Plasticity

The molecular mechanisms of synaptic transmission have been intensely studied in recent decades, resulting in the functional characterization of many synaptic proteins involved in vesicle docking, priming, fusion and recycling. These includes the SNARE proteins, synaptotagmin, regulatory proteins, such as RAB3a, Munc13, Munc18, tomosyn, and active zone proteins, such as Piccolo, Bassoon and RIM1a, as well as several structural and endocytotic proteins (Dresbach et al., 2001; Südhof, 2013; Rizo, 2018; Chanaday et al., 2019). In many cases, the order of interactions involving these proteins, as well as their roles at different stages of the synaptic vesicle cycle, have been extensively described (Brunger et al., 2019). However, the exact contribution of these proteins to synaptic plasticity is still poorly understood. Several second messengers, such as calcium (Ca^2+^) and cyclic adenosine mono-phosphate (cAMP), are known to produce short- and long-term changes in vesicle release probability (P_r_), the number of release sites, and the clustering of calcium channels at the presynaptic terminal. However, whereas the role of Ca^2+^ in regulating both pre- and postsynaptic processes has been described in detail (Zucker, 1999; Schneggenburger and Neher, 2005; Lisman et al., 2007; Kavalali, 2020) the identities of cAMP downstream effectors, especially in the presynaptic terminal, are still largely unknown.

cAMP is a ubiquitous second-messenger found in the three domains of life, namely Eukarya, Bacteria and Archaea (Gehring, 2010; Kalia et al., 2013). A direct link between cAMP, synaptic transmission and potentiation was first demonstrated in a seminal work showing that exposing sensory neurons of the sea mollusk *Aplysia* to cAMP molecules resulted in increased neurotransmitter release, in turn affecting a short-term memory process known as sensitization (Cedar et al., 1972; Klein and Kandel, 1980; Schacher et al., 1988; Kandel et al., 2000). Later studies extended the connection between cAMP and synaptic plasticity to other model organisms, such as *Drosophila* (Davis et al., 1998) and mice (Nguyen and Kandel, 1997; Wong et al., 1999), where cAMP exposure was also shown to lead to an increase in P_r_ (Ariel et al., 2013; Fukaya et al., 2021), vesicle redistribution (Orlando et al., 2021) and changes in Ca^2+^ channel clustering (Midorikawa and Sakaba, 2017).

Until recently, research into the molecular cascades activated by cAMP focused on a single downstream effector—protein kinase A (PKA) (Corbin and Krebs, 1969). PKA is a tetrameric enzyme consisting of two regulatory and two catalytic subunits (Bauman and Scott, 2002; Taylor et al., 2012). Binding of cAMP to the PKA complex results in detachment of the regulatory subunits from the catalytic subunits, thereby removing inhibition from the latter (Turnham and Scott, 2016; Mucignat-Caretta and Caretta, 2020). Subsequently, the catalytic subunits of PKA are able to phosphorylate a myriad of protein targets, thus modifying their functions (Waltereit and Weller, 2003; Kandel, 2012). In *Aplysia*, injection of the PKA catalytic subunits yielded similar effects to those observed following injection of cAMP alone (Castellucci et al., 1980), leading to a prevailing consensus that PKA is the principal, and perhaps sole downstream effector of cAMP. In the mammalian brain, the hippocampal mossy fiber (MF) synapse was found to be particularly prone to cAMP-mediated regulation of neurotransmitter release, as will be discussed in detail below.

## The Mossy Fibers Pathway, Cyclic Adenosine Mono-Phosphate and Protein Kinase A-Dependent Plasticity

**FIGURE 1:**
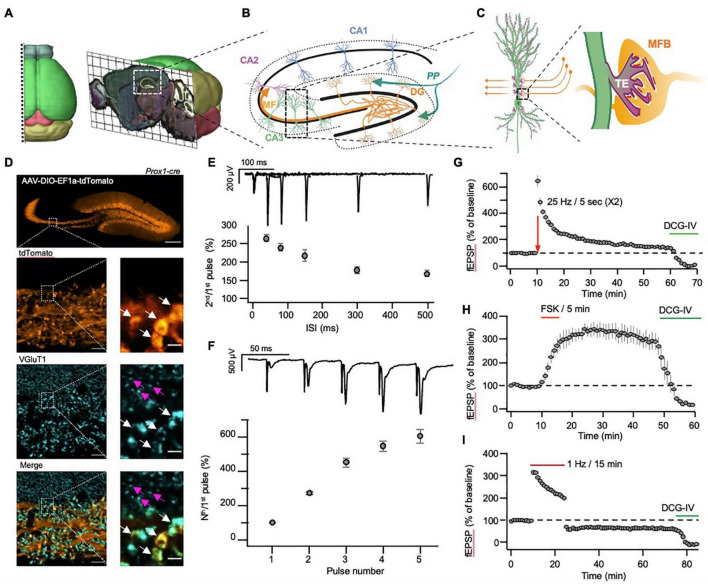
Morphological and physiological properties of the hippocampal mossy fiber pathway. **(A)** Three-dimensional visualization of the anatomical location of the dorsal hippocampus in mouse brain. **(B)** Schematic representation of hippocampal sub-regions, with emphasis on the input and output to and from the DG. **(C)** Schematic representation of the unique anatomical and morphological structure of the MF-CA3 synapse. Insert: mossy fiber bouton (MFB, orange) and postsynaptic thorny excrescence (TE, light green). **(D)** A representative confocal image of the hippocampus following injection of AAV-DIO-EF1a-tdTomato into the DG of a Prox1-cre transgenic mouse (top). The images below show the MF tract at higher magnification (tdTomato, orange), following immunolabeling for VGluT1 (VGluT1, cyan), and demonstrate the size differences between the large MF terminals (white arrows) and the small S.R. terminals (magenta arrows). Scale bars represent 200, 10 and 2 μm for the top, right column and left column images, respectively. **(E,F)** MF-CA3 short-term plasticity demonstrated by measurements of paired-pulse **(E)** and a high-frequency burst **(F)** stimulation, delivered electrically to the DG while recording fEPSPs from the *S.L.* **(G,H)** MF-CA3 long-term plasticity demonstrated by measurements of FSK- **(G)** and tetanus- **(H)** induced potentiation, with subsequent application of DCG-IV, blocking synaptic transmission. **(I).** MF-CA3 long-term plasticity (LTD) following a prolonged low-frequency stimulation, with subsequent application of DCG-IV, blocking synaptic transmission. Images in **(A)** were adapted from the Allen institute’s Brain Explorer 2 (http://mouse.brain-map.org/static/brainexplorer).

The hippocampus is a central cortical structure in the mammalian brain known to mediate key mnemonic and cognitive functions. The hippocampus is classically divided into three unidirectional pathways, collectively known as the trisynaptic circuit. According to this notion of hippocampal information flow, neuronal activity originating in the adjacent entorhinal cortex (EC) is relayed via the perforant pathway (PP), primarily to the hippocampal dentate gyrus (DG), where it is processed and relayed to CA3 and CA2 pyramidal neurons via the mossy fibers (MF) pathway. From these regions, which are internally connected in an auto-associative network, the information is delivered almost exclusively to CA1 pyramidal neurons via Schaffer’s collaterals (SC), which finally redistribute the processed signals across cortical and sub-cortical regions (Figures 1A,B; Lieberman, 1965; Witter, 2007). The MF synapse, corresponding to the second synapse in this circuit, is generally considered to be an important locus for the formation, storage and retrieval of contextual and episodic memories in mammals (Lieberman, 1965; Neves et al., 2008; Lisman et al., 2017), through a computational process termed “pattern separation” (Leutgeb et al., 2007; Schmidt et al., 2012; Rolls, 2013).

The MF pathway is characterized by non-myelinated axons that originate in dentate gyrus granule cells (DGCs) and travel immediately above and below the CA3 *Stratum Pyramidale (S.P.)*, forming the *Stratum Lucidum (S.L.).* There, each axon forms about a dozen enormous synapses (up to several micrometers in diameter (Rollenhagen et al., 2007), termed mossy fiber boutons (MFB), onto the large proximal dendritic spines of CA3 neurons termed thorny excrescences (TEs) (Amaral and Dent, 1981; Figures 1C,D). A single large MFB contains 25 active zones on average and harbors some 16,000 synaptic vesicles, of which only about 600 are located within 60 nm from the AZ and are considered part of the readily releasable pool of vesicles, while an additional 4,000 vesicles are found at a short distance of 200 nm from the AZ and are considered part of the recycling pool (Hallermann et al., 2003; Rollenhagen et al., 2007). Though it has been speculated that this organization might be essential for supporting the various plasticity processes taking place at the MFB (Rollenhagen et al., 2007), the reason for such extreme redundancy of synaptic vesicles remains unclear.

In contrast to its prominent size, the MF synapse is characterized by a very low basal P_r_ (Jonas et al., 1993) and as a result, following a single action potential (AP) elicits weak excitatory post-synaptic potentials (EPSPs) in CA3 neurons (Lysetskiy et al., 2005). However, following a short train of high-frequency APs, the accumulation of Ca^2+^ in the MF synapse produces a dramatic increase in P_r_, manifested as robust short-term synaptic facilitation (Figures 1E,F). This facilitation, together with the strategic location of the synapse in proximity to the CA3 somata, and its multiple release sites, allow a single MF synapse to elicit APs in its postsynaptic target following a short train of APs (Henze et al., 2000; Evstratova and Tóth, 2014; Chamberland et al., 2018). This trait has led researchers to describe the MF synapse as a “conditional detonator” or a “high-pass filter,” due to the tendency of the synapse to selectively propagate high-frequency activity patterns (Pelkey and McBain, 2005; Vyleta et al., 2016).

While other hippocampal synapses have been shown to display primarily postsynaptic N-methyl-D-aspartate receptor (NMDAR)-dependent long-term potentiation (LTP) (Bliss and Collingridge, 2013), MF synapses are characterized by NMDAR-independent LTP (Zalutsky and Nicoll, 1990; Johnston et al., 1992; Huang et al., 1994; Salin et al., 1996b; Castillo, 2012). Long-term potentiation in the MF synapse (MF-LTP, Figure 1G) manifests as a long-term increase in the presynaptic P_r_ and is mediated by cAMP, evident by the robust potentiation observed also following application of the adenylyl cyclase (AC) agonist forskolin (FSK, Figure 1H; Weisskopf et al., 1994; Salin et al., 1996b; Villacres et al., 1998; Castillo, 2012). Using pharmacological tools that control cAMP levels, it was demonstrated that both cAMP and PKA are important for the induction and maintenance of MF-LTP (Huang et al., 1994; Weisskopf et al., 1994). These studies were followed by genetic perturbation of PKA subunits (Huang et al., 1995) that provided genetic evidence for the involvement of PKA in MF-LTP.

Like the MF-synapse, several other synapses in the mammalian brain display NMDAR independent presynaptic forms of LTP (Yang and Calakos, 2013). These include corticothalamic (Castro-Alamancos and Calcagnotto, 1999), thalamocortical (Andrade-Talavera et al., 2013), cortical interneuron (Chen et al., 2009; Sarihi et al., 2012) and subiculo-cortical synapses (Behr et al., 2009), as well as synapses of cerebellar parallel fibers (Salin et al., 1996a; Chen and Regehr, 1997; Bender et al., 2009), and several different inputs to the lateral amygdala (López de Armentia and Sah, 2007; Fourcaudot et al., 2008). However, due to vast morphological and molecular differences between these synapses and the MF, it remains to be determined exactly how similar are the molecular mechanisms that underlie their presynaptic LTP.

In addition to MF-LTP, which is induced by a short train of high-frequency activity, long-term depression in the MF synapse (MF-LTD) can also be elicited, by applying a prolonged (15 min) low-frequency stimulation (Figure 1I). Like MF-LTP, MF-LTD is also NMDAR-independent, however, it is mediated by a reduction in cAMP levels and is manifested as a decrease in P_r_ (Tzounopoulos et al., 1998; Kobayashi, 2010). MF-LTD induction can be blocked by the metabotropic glutamate receptor (mGluR) antagonist MCPG (Fitzjohn et al., 1998) or *mGluR2/3* KO (Lyon et al., 2011). At the same time, application of the mGluR2/3 agonist DCG-IV completely blocks MF synaptic transmission synapses (Figures 1G–I), while having little to no effect on other hippocampal synapses (Yoshino et al., 1996; Kamiya and Ozawa, 1999). Since mGluR2/3 inhibits cAMP synthesis, it can be inferred that bidirectional changes in cAMP concentration control P_r_ at the MF synapse and can lead to LTP or LTD, depending on the direction of the change.

FSK is one of the main pharmacological tools for elevating cAMP levels that leads to synaptic potentiation. However, FSK affects both the presynaptic terminal and the postsynaptic cells, in addition to astrocytes, and is, therefore, not specific for the presynaptic terminal. New recently implemented tools, such as photoactivated adenylyl cyclase bPAC from the bacteria Beggiatoa (Stierl et al., 2011; Raffelberg et al., 2013) or pharmacogenetic tools, like Designer Receptors Exclusively Activated by Designer Drugs DREADDs (Roth, 2016; Campbell and Marchant, 2018), now enable control of cAMP levels with substantially better cellular, spatial and temporal precision than possible using pharmacological agents, such as FSK. Recently, photoactivation of a synaptically-translocated bPAC, termed SynaptoPAC, selectively in MF synapses, enabled cAMP synthesis with physiological kinetics and led to long-term changes in P_r_, mimicking the effects of tetanus-induced LTP (Kees et al., 2021; Oldani et al., 2021). Such tools, together with new cAMP sensors (Odaka et al., 2014; Harada et al., 2017; Ohta et al., 2018; Linghu et al., 2020) will allow better understanding of the effects of cAMP on synaptic physiology at MF synapses.

MF synapses are characterized by additional forms of plasticity that should be briefly mentioned. Although MF-LTP is NMDA-independent, there are indications that presynaptic NMDA receptors support a different form of LTP in the MF synapse, which is dependent on Protein Kinase C (PKC) (Kwon and Castillo, 2008; Lituma et al., 2021). Furthermore, it should be noted that in addition to the large presynaptic MF terminal on pyramidal CA3 neurons, MF axons also innervate *S.L.* interneurons (SLINs) by small *en-passant* varicosities, also known as filopodial extensions (Acsády et al., 1998). These synapses on interneurons contribute to feed-forward inhibition and exhibit different forms of synaptic plasticity mediated by mGluR7 in a process only partially mediated by cAMP (Toth et al., 2000; Pelkey et al., 2008). MF-SLIN synapses also undergo cAMP-dependent plasticity changes, albeit in a unique way. These synapses reverse their polarity in an activity-dependent manner, with the internalization of mGluR7s serving as the switch between two states. In response to high frequency stimulation, naïve-state MF-IN synapses undergo cAMP-independent presynaptic LTD, whereas synapses that have internalized mGluR7s (LTD, resulting from previous high-frequency stimulation) undergo cAMP-dependent presynaptic LTP (Pelkey et al., 2008). This mechanism might allow initial bursts of APs to propagate uninhibited to CA3, yet prevents subsequent bursts from generating runaway excitation (Pelkey et al., 2008).

In addition, most previous research suggests that postsynaptic depolarization is not necessary for MF synapse LTP induction, although under certain conditions, such depolarization could still exert influence over presynaptic physiology (Castillo et al., 2012; Makani et al., 2021; Vandael et al., 2021). This implies that synchronous activation of pre- and postsynaptic neurons is not necessarily a prerequisite for this form of LTP, suggesting that MF synapse LTP is not Hebbian in nature. Together, these unique properties of the MF synapse are likely to support roles served by the hippocampus, such as the suggested role of MFs in spatial orientation through a mnemonic function known as pattern separation (Yassa and Stark, 2011; Schmidt et al., 2012; Rolls, 2013).

## The Role of Ca^2+^ and Adenylyl Cyclase 1 in Mossy Fibers Plasticity

As in most other synapses, vesicle release at the MF terminal depends on Ca^2+^ influx through voltage-dependent calcium channels (VGCC), such as the P/Q-, N-, L-, and R-type calcium channels (Li et al., 2007; Vyleta and Jonas, 2014; Shin et al., 2018). In addition, the activation of presynaptic NMDA receptors during high frequency activity (Carta et al., 2018; Lituma et al., 2021) and release of Ca^2+^ from internal stores (Lauri et al., 2003; Scott and Rusakov, 2006) also contribute to Ca^2+^ dynamics at the MF synapse. These dynamic changes in presynaptic calcium drive MF short- and long-term synaptic plasticity (Castillo et al., 1994; Regehr et al., 1994; Kapur et al., 1998; Breustedt et al., 2003; Pelkey et al., 2006; Li et al., 2007; Vyleta and Jonas, 2014). In a mature MFB, it was demonstrated that synaptic vesicles are only loosely coupled to Ca^2+^ channels, allowing for a highly dynamic P_r_ range (Vyleta and Jonas, 2014; Böhme et al., 2018; Brockmann et al., 2019; Orlando et al., 2021). Changes in distances between calcium channels and the synaptic machinery, in channel density, and/or in concentrations of endogenous calcium buffers can enable rapid and dynamic modulation of synaptic strength, as discussed below.

**FIGURE 2:**
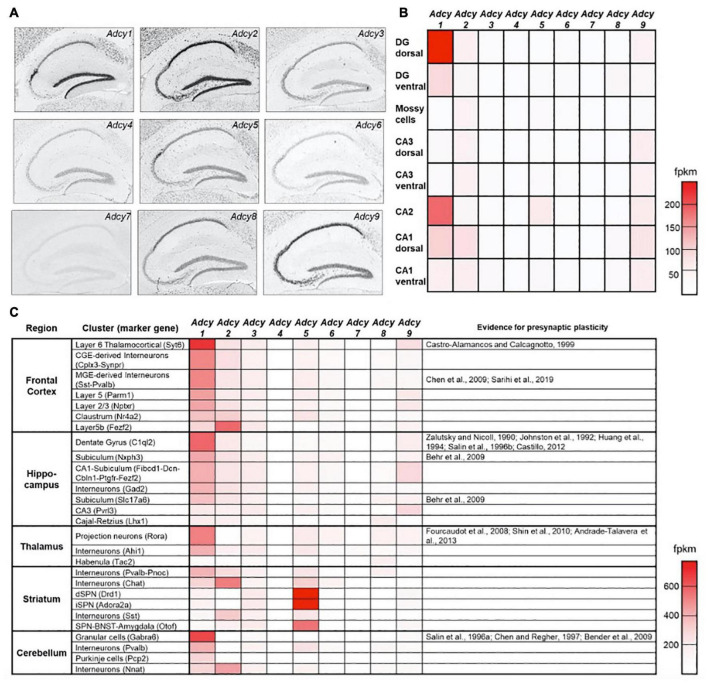
Expression of AC isoforms across hippocampal sub-regions. **(A)** Distribution pattern of the ten AC isoforms in the hippocampus of adult mice following *in situ* hybridization (Lein et al., 2007). **(B)** A heat map showing the relative mRNA expression levels of *Adcy* isoforms 1–9 across different hippocampal sub-regions. fpkm—fragments per kilobase of transcript per million mapped reads (Cembrowski et al., 2016). **(C)** as in **(B)**, a heat map showing relative *Adcy* isoform 1–9 mRNA expression levels for the main cell clusters across several central brain regions. The right-most column specifies for which cell clusters evidence supports the existence of presynaptic LTP (Saunders et al., 2018). Data in **(A)** were adapted from the Allen institutes ISH brain atlas (http://mouse.brain-map.org/), data in **(B)** were adapted from hipposeq.janelia.org/and data in **(C)** were adapted from Dropviz.org.

In addition to its direct effects on vesicle fusion and neurotransmitter release, Ca^2+^ also exerts a powerful effect on MF synaptic strength through the Ca^2+^ sensor calmodulin and activation of AC, leading to increased levels of cAMP. Numerous studies have pointed to cAMP as the primary mediator of MF-LTP, given how the application of the potent AC agonist FSK or of cAMP analogs, such as Sp-8-CPT-cAMPs, elicit a strong and sustained increase in the basal P_r_ (Weisskopf et al., 1994; Lonart et al., 1998; Tzounopoulos et al., 1998; Wang et al., 2003; Kaeser-Woo et al., 2013; Hashimotodani et al., 2017). In the mammalian brain, ten different isoforms of the *Adcy* gene encode for ten distinct AC enzymes (AC1-10). These are classified primarily according to their main upstream activator (Hanoune and Defer, 2001) and are differentially distributed across brain regions and cell types (Sanabra and Mengod, 2011). In the mouse hippocampus, *Adcy* isoforms 1 and 2 are strongly and selectively expressed in the DG but not in CA3 neurons (Figures 2A,B), and while KO of *Adcy8* was shown to affect MF synaptic plasticity (Wang et al., 2003), comparatively low mRNA levels in the DG (Figures 2A,B) suggest that further experiments are needed to validate these results. Of the two most abundant AC isoforms in the DG, AC1 is known to be activated by an increase in Ca^2+^ and its downstream effector calmodulin (Hanoune and Defer, 2001), whereas AC2 is largely Ca^2+^-insensitive and is instead activated by PKC and the Gq protein-associated βγ subunit complex (Hanoune and Defer, 2001; Willoughby and Cooper, 2007; Halls and Cooper, 2011). Alongside these basic properties, additional evidence further supports a central role for AC1 in the MF synapse. First, AC1 is inhibited by the G_*i*_ cascade, such as that associated with mGluR2/3, leading to a reduction in cAMP levels (Sadana and Dessauer, 2009), an effect which potentially underlies the complete silencing of MF synaptic transmission following application of DCG-IV. Furthermore, *Adcy1* KO mice were found to exhibit impaired MF-LTP but not PP-LTP and while MF short-term plasticity and FSK-induced potentiation were not altered in *Adcy1* KO mice (Villacres et al., 1998; Table 1), this effect could have still been mediated by other AC isoforms, being that FSK is not a selective AC1 agonist. Last, immunolabeling of AC1 in the hippocampus revealed it to be enriched in the hilus and mossy fibers (Conti et al., 2007), implying that AC1 is preferentially trafficked to MF synaptic domains.

**TABLE 1:** The effects of manipulation of proteins on hippocampal mossy fiber synaptic plasticity.

KO/KD	STP	LTP	Fsk-induced potentiation (FIP)	Citations
Rab3A	Not affected	Abolished	Not affected	Castillo et al., 1997
RIM1a	Not affected	Abolished	Not affected	Castillo et al., 2002
Rabphilin	Not affected	No effect	Not determined	Schlüter et al., 1999
Synapsin	Not determined	No effect	Not affected	Spillane et al., 1995
Synaptotagmin-12	Not affected	Abolished	Impaired	Kaeser-Woo et al., 2013
Tomosyn	Impaired	Abolished	Impaired	Ben-Simon et al., 2015
PKAα	Not determined	Impaired	Not determined	De Lecea et al., 1998
AKAP7	Not determined	Abolished	Not determined	Jones et al., 2016
Epac2	Not affected	Abolished	Impaired	Fernandes et al., 2015
AC1	Not affected	Impaired	Not affected	Villacres et al., 1998

Together, these observations suggest that AC1 is the dominant isoform mediating Ca^2+^ dependent increases, or mGluR2/3-dependent decreases in cAMP levels, to control MF synaptic plasticity. Interestingly, the distribution of AC1 mRNA across brain regions reveals strong correlation between cell types in which *Adcy1* is enriched, with such synapses having been previously shown to express a presynaptic form of LTP (Figure 2C). This correlation can potentially support the notion that shared molecular mechanisms, driven by up-stream AC1 activation, underlies presynaptic LTP across cell types. In the hippocampus, this conjuncture could apply to neurons of the CA2 sub-region, in which *Adcy1* is also enriched. While previous studies suggested that CA2 neurons do not display any form of postsynaptic plasticity (Zhao et al., 2007), the nature of the presynaptic mechanisms at play in their synapses onto CA1 neurons, corresponding to their principal output (Kohara et al., 2014), have not yet been investigated. Further experiments are required to determine whether cAMP- and PKA-dependent presynaptic LTP can also be induced there, and if so, one can ask what sort of functional relevance this might have on information processing in this pathway.

In addition to the Ca^2+^-mediated activation of AC1, increased cAMP levels could also potentially arise from direct activation of AC2 and AC9 through the alpha subunit of G_s_ protein-coupled receptors (Hanoune and Defer, 2001), such as group 1 serotonin receptors (Hamblin et al., 1998), the beta sub-class of noradrenaline receptors (Johnson, 2006) and dopamine D1-like receptors (Girault and Greengard, 2004). In contrast, other G_i_ protein-coupled neuromodulator receptors, such as group 1 and 5 serotonin receptors, the alpha2 sub-class of noradrenergic receptors and the dopamine D2 receptor, would have an opposite effect on cAMP production, potentially supporting MF-LTD. It has previously been shown that neuromodulators can indeed exert effects on MF synaptic transmission in various manners (Yang and Calakos, 2013; Nozaki et al., 2016; Kobayashi et al., 2020). This contradictory mode of action of different receptors in response to the same ligand and their differential expression patterns across hippocampal sub-regions make it difficult to determine what would be the net effect of each receptor on synaptic transmission and plasticity. In addition, it has yet to be shown where these receptors are trafficked within the cells and whether they are enriched in presynaptic domains. Such knowledge is essential for determining whether neuromodulators could have a direct effect on neurotransmitter release at the MF-CA3 synapse, or whether the observed effects of neuromodulator application during recordings arise from changes in DGC somatic excitability or from various influences on CA3 postsynaptic domains. The use of specific cAMP sensors (Odaka et al., 2014; Harada et al., 2017; Ohta et al., 2018; Linghu et al., 2020) that can be targeted to the synapse will allow characterization of the spatiotemporal distribution of cMAP and can clarify the above debates.

## Molecular Mechanisms of Protein Kinase A-Dependent Mossy Fibers Plasticity

**FIGURE 3:**
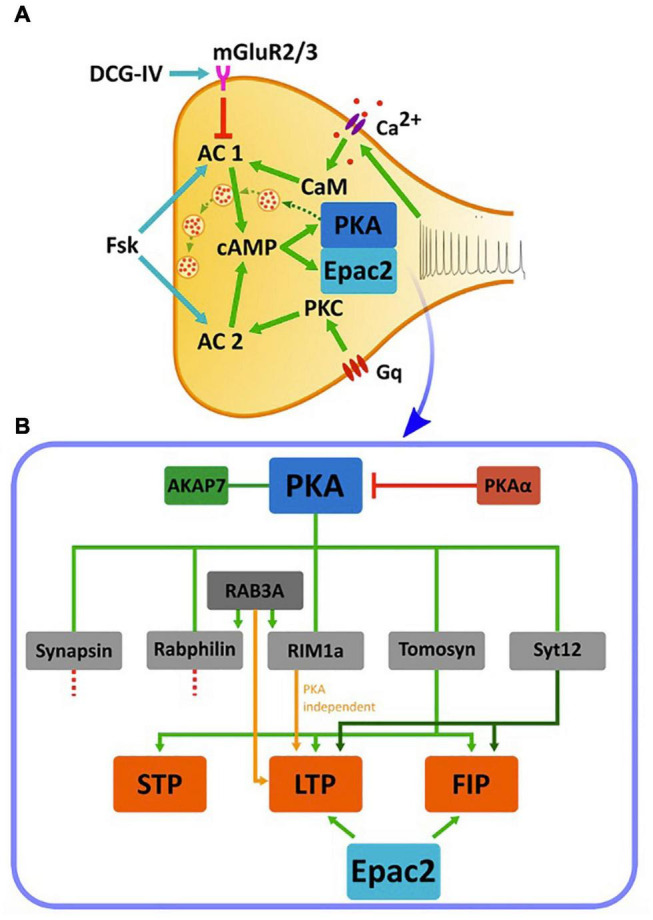
cAMP cascades and cAMP-dependent synaptic plasticity in the MF synapse. **(A)** Cascades of activation of cAMP, PKA, and Epac2 in the MF terminal. A train of action potentials arriving at the MF synapse triggers a calcium influx through voltage-dependent calcium channels and subsequent activation of CaM that activates cAMP synthesis by AC1. AC1 acts on PKA or Epac2 to up regulate synaptic transmission (dashed green arrows between vesicles). Application of FSK activates both AC1 and AC2 and elevates cAMP. AC2 is also activated by PKC following the activation by a G-coupled protein. Application of DCG-IV, an agonist to mGluR2/3, leads to inhibition of AC1. Green arrows: activation, red arrow: inhibition, blue arrows: application of pharmacological reagents. **(B)** Downstream PKA cascades and their relation to synaptic plasticity. PKA activity is dependent on the anchoring protein AKAP7, and is inhibited by the negative regulator PKAα. PKA, in turn, phosphorylates multiple proteins including synapsin, rabphilin, tomosyn-1, synaptotagmin12 (Syt12) and RIM1a. Of these, deletion of synapsin and rabphilin does not impair MF synaptic plasticity. Ablation of RAB3A, although not a direct PKA target, and RIM1a abolishes MF-LTP but does not affect STP and FSK-induced potentiation (FIP). Synaptotagmin-12 KO abolishes MF-LTP and impairs FSK-induced potentiation but do not affect STP, while deletion of tomosyn-1 abolishes MF-LTP, impairs FSK-induced potentiation and also affects STP. Epac2 manipulation also abolishes LTP and impairs FSK-induced potentiation. STP- short-term potentiation, LTP- long-term potentiation, FIP- FSK-induced potentiation. Orange arrow represent PKA-independent pathway, Green arrows shades represent different pathways.

As mentioned above, MF-LTP depends on cAMP levels, with this effect having largely been attributed to downstream activation of PKA. This assessment was further supported by the observation that LTP is absent following KO of the PKA catalytic subunit-encoding gene (Huang et al., 1995) and that application of KT5720, a selective blocker of the PKA catalytic subunit, prevents MF-LTP (Weisskopf et al., 1994). In addition, it was suggested that FSK-induced potentiation interacts with LTP, as the two processes were shown to mutually occlude one another (Weisskopf et al., 1994). These findings led to the suggestion that activation of PKA is an essential step in MF-LTP. Accordingly, research conducted in recent decades identified several putative synaptic PKA targets thought to be involved in cAMP-dependent synaptic plasticity at MF synapses. These include RAB3a-interacting molecule 1a (RIM1a) and rabphilin, both acting through the vesicular GTPase Ras-related protein Rab-3A (RAB3a) (Takai et al., 1996; Wang et al., 2001), as well as synapsin (Bykhovskaia, 2011), synaptotagmin-12 (Maximov et al., 2007), and tomosyn-1 (Ashery et al., 2009). The involvement of these proteins in cAMP-dependent plasticity was mainly studied by examining the effects of deletion or mutations in each of the encoding genes on short-term plasticity (STP), FSK-induced potentiation, LTP and LTD (Table 1 and Figure 3).

Earlier efforts paid substantial attention to the small vesicular GTPase RAB3a, which is seemingly involved in vesicle mobilization and fusion (Geppert et al., 1997; Lonart and Südhof, 1998). While RAB3a is not considered a putative PKA target, it is known to be regulated by other PKA targets, such as RIM1a and rabphilin and has, therefore, been linked to the PKA-mediated cascade. *RAB3a* KO was shown to block LTP, impair LTD formation in MF synapse and led to memory impairment (D’Adamo et al., 2004), although neither STP nor FSK-induced potentiation were affected (Castillo et al., 1997). These effects were thought to be the result of an enhanced effect of Ca^2+^ on the secretory apparatus (Lonart et al., 1998), although the precise mechanism involved was not identified.

RIM1a is an active zone protein and putative PKA target (Wang et al., 1997; Castillo et al., 2002; Lonart et al., 2003). Similar to what was seen in the absence of RAB3a, KO of *RIM1a* also prevented MF-LTP but not FSK-induced potentiation or STP (Castillo et al., 2002; Figure 3 and Table 1), suggesting a direct link between PKA, RIM1a, RAB3a, and MF-LTP. However, this effect appears to be PKA-independent, as RIM1a*S*^413A^, an isoform that carries a point mutation preventing RIM1a phosphorylation by PKA, was found to have no impact on either short- or long-term plasticity, nor on behavior (Kaeser et al., 2008). Hence, despite the dependence of LTP on RIM1a, the link through PKA is less defined and suggests that PKA operates either through a different mechanism, or via yet another unidentified parallel pathway/s.

Rabphilin, another RAB3a effector, regulates vesicle priming through interactions with RAB3a and the SNARE protein SNAP-25, with the former occurring in a GTP-dependent manner (Tsuboi and Fukuda, 2005; Deák et al., 2006). Rabphilin was found to be phosphorylated following application of FSK in the MF but not in the SC synapse (Lonart and Südhof, 1998), highlighting that significant differences exist in the molecular mechanisms underlying synaptic plasticity between these different hippocampal regions. However, KO of the *rph3a* gene encoding for rabphilin did not change either short-term facilitation or MF-LTP at the MF terminal (Schlüter et al., 1999; Figure 3 and Table 1).

In addition to the RAB3a cascade, synapsin was considered as another candidate PKA downstream effector. Synapsins are among the most abundant synaptic proteins that are known to be phosphorylated by PKA, and are involved in both synaptic transmission and plasticity (De Camilli and Greengard, 1986; Gitler et al., 2004). Accordingly, synapsins were one of the first synaptic protein families to be examined as targets for cAMP and PKA-dependent plasticity at the MF. Synapsin binds synaptic vesicles and tethers them to the cytoskeleton, yet can release these vesicles following phosphorylation by PKA, enabling their translocation to the active zone in preparation for exocytosis (De Camilli et al., 1983; Spillane et al., 1995). A triple KO of all three synapsin isoform-encoding genes led to impaired plasticity in primary hippocampal neuronal cultures, with such changes being associated with PKA activity (Cheng et al., 2018). However, in the MF pathway of acute hippocampal slices, a double KO of the *syn*1 and *syn*2 genes, encoding the main isoforms expressed in CNS neurons, did not alter cAMP-dependent LTP or FSK-induced potentiation (Figure 3B and Table 1; Spillane et al., 1995). While further research is required to reconcile these contradicting results, these findings suggest that phosphorylation of synapsin1 and synapsin2 by PKA is unlikely to be required for cAMP-dependent MF synaptic plasticity.

More recent studies have examined other synaptic proteins thought to be involved in PKA-dependent LTP at the MF synapse. Synaptotagmin-12 differs from other synaptotagmins in that it does not bind Ca^2+^ (Wolfes and Dean, 2020). However, synaptotagmin-12 is phosphorylated by PKA and is involved in the regulation of synaptic vesicles fusion and P_r_ (Maximov et al., 2007). Synaptotagmin-12^S97A^ mutant mice, in which the site of PKA-mediated phosphorylation was mutated, displayed impaired MF-LTP and FSK-induced potentiation, although their STP remained unaltered (Kaeser-Woo et al., 2013). Consequently, synaptotagmin-12 can be regarded as the first protein shown to affect both LTP and FSK-induced potentiation in a PKA-dependent manner.

Tomosyn is a PKA-dependent negative regulator of P_r_ and its over-expression leads to an inhibition of vesicle priming, while knockdown, knockout, or mutations in the *tomosyn-1*-encoding gene led to an enhancement of vesicle fusion (Yizhar et al., 2004; Chevlet et al., 2006; Gracheva et al., 2006; McEwen et al., 2006; Ben-Simon et al., 2015). Tomosyn-1, which was shown to be enriched in the MF pathway (Barak et al., 2010, 2013), can be phosphorylated by PKA at Ser-724 (Baba et al., 2005) and its acute down-regulation in the MF synapse leads to reductions in both LTP and FSK-induced potentiation (Ben-Simon et al., 2015). Interestingly, this manipulation also strongly reduced MF facilitation and STP. These effects are likely the result of an increase in the basal P_r_ following down-regulation of tomosyn-1, which also led to occlusion of both potentiation and facilitation. Although acute KD of tomosyn reduced both LTP and FSK-induced potentiation (Ben-Simon et al., 2015), a direct link between PKA-driven tomosyn-1 phosphorylation and synaptic plasticity has yet to be demonstrated.

Although FSK-induced potentiation and MF-LTP are thought to be interlinked and are driven by cAMP elevation (Weisskopf et al., 1994), deletion of most PKA-dependent synaptic proteins abolished only MF-LTP and not FSK-induced potentiation. This could suggest the presence of additional parallel cAMP-dependent pathways that are not fully PKA-dependent. It is also possible that high frequency stimulation-induced LTP and FSK affect downstream events with different cAMP-mediated dynamics, which would differently affect MF-LTP and FSK-induced potentiation. However, the same does not apply to the loss of synaptic proteins, such as of tomosyn-1 or synaptotagmin-12, which impaired both MF-LTP and FSK-induced potentiation (Kaeser-Woo et al., 2013; Ben-Simon et al., 2015). It is reasonable to assume that the impact of the loss of tomosyn-1 on MF-LTP and FSK-induced potentiation can be explained through an increase in basal P_r_ that is further translated into a reduction in synaptic potentiation. What is clear is that further studies are needed to characterize the exact contributions of these and other synaptic proteins and pathways to the cAMP dependent plasticity of the MF.

Additional proteins that interact with PKA, like PKAα and AKAP7, have also been shown to affect MF plasticity. PKAα is a PKA inhibitor whose activity decreases following synaptic stimulation, leading to a relief of PKA inhibition during neuronal activity. Moreover, blocking this pathway with anti-sense oligonucleotides to *PKA*α resulted in elimination of MF-LTP in the synapse (De Lecea et al., 1998). AKAP7 is a PKA-anchoring protein also essential for the function of PKA. Ablation of AKAP7 results in even more pronounced effects than those seen with PKAα inhibition, specifically, not only the elimination of MF-LTP but also various measurable behavioral deficits (Huang et al., 1995; Jones et al., 2016; Figure 3B and Table 1).

## Cyclic Adenosine Monophosphate and Restructuring of Synaptic Release Sites

Recent results obtained with isolated MF terminals indicate that cAMP can directly affect P_r_ via regulation of Ca^2+^ signaling (Midorikawa and Sakaba, 2017; Fukaya et al., 2021). Applying cAMP to isolated MF terminals increased P_r_, although the number of vesicles in the readily releasable pool and replenishment of this pool following depletion remained unchanged. It has been suggested that this increase in P_r_ is associated with changes in the physical coupling between of P/Q-type Ca^2+^ channels and readily releasable vesicles (Midorikawa and Sakaba, 2017), which can alter the synaptic properties at the terminal (Bucurenciu et al., 2008; Vyleta and Jonas, 2014). As such, following an action potential, Ca^2+^ concentrations near release sites are expected to be higher, thereby leading to synaptic potentiation. Such molecular rearrangements were recently shown to occur within 5–10 min following FSK application (Fukaya et al., 2021).

A recent paper showed that FSK-induced potentiation was associated with fast remodeling of MF synapse presynaptic ultrastructure. However, the distance between P/Q-type calcium channels and Munc13–1 as a proxy for the release machinery was not altered and was found to be 65 nm (Orlando et al., 2021). On the other hand, FSK-induced potentiation was associated with synaptic vesicle accumulation at active zones, increases in the numbers of docked and tethered vesicles and an overall increase in the number of active AZs. In addition, vesicles were more dispersed, possibly mediated by PKA phosphorylation of synapsin (Sihra et al., 1989; Pechstein et al., 2020), allowing for the mobilization of vesicles into the readily releasable vesicle pool. A similar increase in the number of docked vesicles was recently suggested to take place following high-frequency stimulation of MF synapses (Vandael et al., 2020) and was referred as a “pool engram,” enabling post-tetanic potentiation. Reorganization of active zone and changes in the recycling vesicle pool also occurs after FSK-induced LTP at CA3-CA1 synapses and might represent a basic mechanism of presynaptic LTP in central synaptic synapses (Rey et al., 2020) and in the *Drosophila* neuromuscular junction (Weyhersmüller et al., 2011). Such processes are likely to be mediated by AZ proteins, such as RIM1a that interacts with Munnc13–1, or Rab3A together with calcium channels (Betz et al., 2001; Schoch et al., 2002; Han et al., 2011; Kaeser et al., 2011; Eggermann et al., 2012), via RIM-BP2 that stabilizes Munc13-1 protein clusters at MF AZs channels (Brockmann et al., 2019), or via synapsin phosphorylation. However, as mentioned above, PKA-mediated phosphorylation of RIM1a was shown to have no effect on MF synaptic transmission (Kaeser et al., 2008), while synapsin KO had no effect on MF-LTP. Additional super-resolution microscopy methods that can inspect organizational changes at the 10–20 nm range can resolve how the release machinery, AZ and calcium channels are restructured following elevations in cAMP levels.

## Alternative Cyclic Adenosine Monophosphate Targets

**FIGURE 4:**
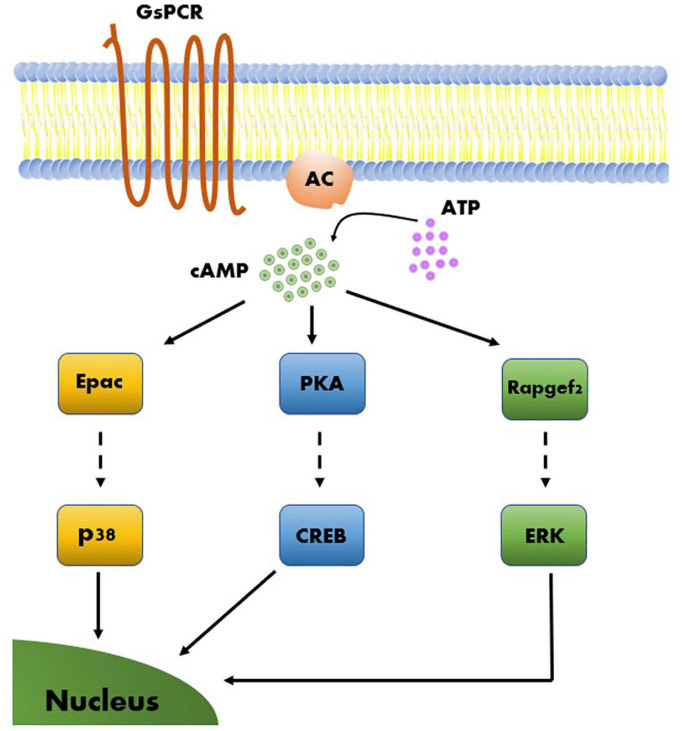
Model of molecular mechanisms of cAMP-dependent LTP. Three cAMP-dependent effectors, Epac, PKA, and Rapgef2, give rise to three parallel molecular pathways. Each of the three cAMP-dependent molecular pathways include a transcription factor that upon activation leads to long-term effects on gene regulation that support synaptic plasticity.

PKA is considered by many to be a master regulator of synaptic plasticity in the hippocampal MF synapse, as well as in many other synapses (Waltereit and Weller, 2003). Consequently, other than the intensive studies conducted on the involvement of PKA targets in MF-LTP, only few studies have considered other possible cAMP-dependent pathways. One such example is a study that linked Epac2, a member of a family of cAMP-dependent guanine nucleotide exchange proteins (GEFs), to synaptic plasticity at the MF synapse (Fernandes et al., 2015). The two cAMP-dependent GEFs, Epac1, and Epac2 (encoded by the *Rapgef3* and *Rapgef4* genes, respectively), were linked to several cAMP-dependent processes (Gloerich and Bos, 2010), and it was further suggested that these proteins are relevant to synaptic plasticity processes (De Rooij et al., 1998; Kawasaki et al., 1998; Fernandes et al., 2015). Epac was shown to activate a MAP-kinase called p38 (Figure 4) in cerebellar and hippocampal neurons and this was correlated with modulation of neuronal excitability (Ster et al., 2007, 2009). In a separate study, several experiments have demonstrated that blocking Epac2 signaling led to reduced levels of p38 phosphorylation (Emery et al., 2014). This suggests that Epac2 responds to cAMP by activating ERK and the small GTPase Rap, as well as its downstream kinase p38-MAPK, thereby bypassing the canonical PKA cascade (Figure 4). Epac2 plays a crucial role in MF-LTP, as KO of the encoding gene led to deficits in MF-LTP and impaired FSK-induced potentiation without affecting basal synaptic properties, as measured by changes in STP (Fernandes et al., 2015; Figure 3B and Table 1).

More recently, an additional member of the RAPGEF family, RapGEF2, was hypothesized to participate in neuronal and synaptic processes. Due to its unconventional cAMP-binding motif, RapGEF2 was long considered not to be a cAMP sensor (Liao et al., 1999; Kuiperij et al., 2003; Kannan et al., 2007). However, more recent studies have shown that a specific RAPGEF2 isoform expressed exclusively in neurons and endocrine cells, termed NCS-Rapgef2, can phosphorylate ERK in a cAMP-dependent manner in these cells (Emery et al., 2013; Figure 4). In neurons, this observed effect was modulated by activation of the dopamine receptor D1 (DRD1), potentially modulating postsynaptic sensitivity in response to dopamine release as well as in a dopamine receptor D1 (DRD1)-dependent manner (Jiang et al., 2017). In addition, KO of *NCS-Rapgef2* in DRD1^+^ medium spiny neurons (MSN) of the nucleus accumbens was shown to have behavioral consequences, resulting in impairments in cocaine-induced locomotor sensitization (CILS) and in conditioned place preference (Jiang et al., 2021). Interestingly, CILS was previously shown to require ERK-dependent DRD1-MSN neuroplasticity (Ferguson et al., 2006; Girault et al., 2007), further supporting the contribution of ERK-dependent pathways and the possible involvement of NCS-RAPGEF2 in neuroplasticity. Still, the involvement of NCS-Rapgef2 in presynaptic plasticity has yet to demonstrated.

Based on current understanding, we can assume the existence of at least three main cAMP-dependent molecular pathways relevant to synaptic plasticity. These can be described according to the proteins directly activated by cAMP, and by the principal kinases or RAPGEFs downstream of these proteins, which execute many of the regulatory functions ascribed to their respective pathways. The three principal pathways are PKA → CREB, Epac → p38 and Rapgef2 → Erk (Figure 4). These pathways can work independently but can also interact and influence one another (Emery et al., 2014, 2016, 2017). Lastly, these molecular pathways can activate plasticity processes that differ in several aspects, such as the locus of plastic changes (e.g., pre- vs. postsynaptic plasticity), resistance to pharmacological compounds, and even associated behavioral processes (Morozov et al., 2003; Fernandes et al., 2015).

## Conclusion

In summary, the presynaptic forms of LTP and LTD that transpire in the hippocampal MF-CA3 synapse are thought to depend on cAMP-PKA-dependent cascades. Various synaptic proteins have been linked to these cascades, and manipulations of several of these proteins, such as RAB3a, RIM1a, synaptotagmin-12, and tomosyn-1, are crucial for signaling along these cascades. Interestingly, manipulations of these proteins mostly impact MF-LTP formation and only in some cases, FSK-induced potentiation or STP. This indicates the strong possibility of the existence of several parallel cAMP-dependent cascades, which are still only partially understood. Mechanistically, elevation of cAMP in the MF synapse involves the rearrangement of synaptic vesicles near release sites and the clustering of calcium channels near the release machinery, which lead to synaptic potentiation. Adopting additional methods that can detect nanometric-level organizational changes in the AZ will help resolve how the release machinery is restructured following elevation in cAMP with more detail. Such changes in synaptic plasticity can be mediated via other PKA targets or via non-canonical PKA-independent but cAMP-dependent pathways, like those involving the RAPGEF family of proteins. Combinations of cell type-specific manipulation of specific proteins, along with synapse-specific manipulation of cAMP by either optogenetic or pharmacogenetic approaches, as well as the ability to measure cAMP levels in response to natural stimulation patterns, will allow a more detailed understanding of the relationship between cAMP and synaptic plasticity at the MF synapse. Such findings could potentially be extrapolated to other synapses, which bear similar physiological hallmarks, to expand our understanding of the links between cellular transcriptomics, synaptic physiology and behavior.

## Author Contributions

All authors contributed to discussion, writing, and editing the manuscript.

## Conflict of Interest

The authors declare that the research was conducted in the absence of any commercial or financial relationships that could be construed as a potential conflict of interest.

## Publisher’s Note

All claims expressed in this article are solely those of the authors and do not necessarily represent those of their affiliated organizations, or those of the publisher, the editors and the reviewers. Any product that may be evaluated in this article, or claim that may be made by its manufacturer, is not guaranteed or endorsed by the publisher.
